# The Psora Theory

**Published:** 1891-03

**Authors:** 


### HOMŒOPATHIC PHYSICIAN,

A MONTHLY JOURNAL OF

HOMŒOPATHIC MATERIA MEDICA AND CLINICAL MEDICINE.

"If our school ever gives up the strict inductive method of Hahnemann, we are lost, and deserve only to be mentioned as a caricature in the history of medicine."—CONSTANTINE HERING.

Vol. XI. MARCH, 1891. No. 3.

### EDITORIAL.

THE PSORA THEORY.—Beside familiarity with the law governing Homœopathy, and the corollaries of that law, there is required of the successful homœopathician powers of observation which should be constantly cultivated. By honestly adhering to our law success in the treatment of the sick is so certain that we are apt to become lax in closely observing much that goes to make more clear the cause of that success. None of the teachings of Hahnemann have received more condemnation than his Psoric theory, and yet but even slight observation will enable him who clearly grasps the master's teachings in respect thereof to put aside all doubt regarding its value, and also to convince those who deny, but who are open to conviction, as to its being true. To him who has given little or no attention to this subject we commend the writings of those whose vigilance has done much in showing the truth of Hahnemann's teachings.

In our last number Dr. Lee called attention to the writings of Dr. P. P. Wells, found throughout this journal. We heartily commend these to the younger followers of Hahnemann particularly, for we know of no articles which make more clear the philosophy of Homœopathy.

We would now direct our readers to some observations of

Grauvogl in connection with the Psoric theory. To many homœopathicians, especially those who are intimate with Hahnemann's *Chronic Diseases*, the facts of Psora and the many ailments due thereto seem so self-evident that they rarely write on the subject. But when the question arises they are ever ready to attest its truth.

Grauvogl first learned from Dr. Reuter, of Nuremberg, that many years' experience had shown him that in those of a psoric tendency there would follow in almost unbroken succession the following characteristics, "provided that up to the last stage no medical aid had been sought:" 1. Gastroses; 2. Catarrhs; 3. Hemorrhoids; 4. Sweat of the feet; 5. Hoarseness; 6. Headache and toothache; 7. Diseases of the eyes; 8. Diseases of the ears; 9. Prurigo of the trunk, Furunculosis; 10. Swelling of the cervical glands; 11. Rheumatism; 12. Swelling of the axillary glands.

For example, if one is found suffering any of the above ailments, there will be established the former presence of those of a preceding number. Dr. Reuter, says Grauvogl, "accordingly shaped his therapeutics in such a manner as to undertake nothing which could disturb the reappearance of the previous numbers." In doing this he was following the teachings of Hahnemann, and thus adopting the only successful method of bringing about a normal condition of system. Here we have a beautiful example of the disappearance of symptoms inversely in the order of their appearance, which has been proved to be a guide showing progress toward a cure in all chronic diseases.

It was found, and every observer can testify to this, that unless symptoms did thus disappear there was evidence of an extension of this peculiar miasm, and instead of improvement there would appear other ailments. It is only the true homœopathician who can appreciate these observations, and it is incumbent upon all to attend to them, else they will never be able to accomplish as much as was achieved by those who in former days laid the foundations for the advance of Homœopathy.

